# Challenges in ITS-based identification of *Cunninghamella bertholletiae* and *Rhizopus microsporus* due to intragenomic ITS sequence heterogeneity

**DOI:** 10.1128/spectrum.03485-25

**Published:** 2026-07-06

**Authors:** Wan-Lin Wu, Pui-Ching Choi, Hsuan-Chen Wang, Ming-I Hsieh, Chi-Jung Wu

**Affiliations:** 1 National Institute of Infectious Diseases and Vaccinology, National Health Research Institutes, Tainan, Taiwan; 2 Division of Infectious Diseases, Department of Internal Medicine, National Cheng Kung University Hospital, College of Medicine, National Cheng Kung University 34912, Tainan, Taiwan; Mycology Laboratory, Wadsworth center, Albany, New York, USA

**Keywords:** *Cunninghamella bertholletiae*, *Cunninghamella echinulata*, D1/D2, ITS, intragenomic heterogeneity, *Rhizopus microsporus*

## Abstract

*Cunninghamella bertholletiae* and *Rhizopus microsporus* are clinically important Mucorales that frequently exhibit mixed peaks in chromatograms from conventional Sanger sequencing of the internal transcribed spacer (ITS) region. Intragenomic ITS heterogeneity has been reported in *R. microsporus* but has not yet been characterized in *Cunninghamella* species. This study investigated ITS patterns in nine *C. bertholletiae*, three *Cunninghamella echinulata*, and two *R. microsporus* isolates collected in Taiwan. The ITS-D1/D2 regions were examined by PCR, cloning, and Sanger sequencing; one *C. bertholletiae* isolate was further studied using PacBio HiFi sequencing. In *C. bertholletiae*, cloning and Sanger sequencing revealed length variation at six homopolymeric tracts ≥ 8 bp within the ITS region, consistent with PacBio findings. *C. echinulata* exhibited none or minimal intragenomic ITS variation. Both *R. microsporus* isolates contained divergent ITS and D1/D2 sequence patterns; one strain showed substantial indels, resulting in intragenomic divergence of 27.4% in ITS and 3.10% in D1/D2. These hybrid ITS sequence patterns were highly similar to those observed in strains from Hong Kong, suggesting that ancient hybridization has been stably inherited. Overall, intragenomic divergence ranged 0.14%–0.55% in *C. bertholletiae*, 0%–0.23% in *C. echinulata*, and 1.19%–27.37% in *R. microsporus* for ITS, and 0%–0.41%, 0%–0.41 %, and 1.43%–3.10% for D1/D2, respectively. In conclusion, our study revealed the patterns of ITS heterogeneity, including length variation in long homopolymeric tracts in *C. bertholletiae* and hybrid ITS patterns in *R. microsporus*, which contribute to Sanger chromatogram ambiguity and pose challenges for ITS-based identification. Although D1/D2 sequencing offers an alternative, its utility may also be affected by intragenomic divergence in *R. microsporus*, highlighting the need for single-copy genes to improve species identification.

**IMPORTANCE:**

C*unninghamella bertholletiae* and *Rhizopus microsporus* are clinically important Mucorales that often yield ambiguous internal transcribed spacer (ITS) chromatograms in Sanger sequencing. We showed that in *C. bertholletiae*, chromatogram ambiguity arises from length variation in six long homopolymeric tracts (≥8 bp), while in *R. microsporus,* it results from hybrid ITS types, as observed in isolates from Hong Kong. Such intragenomic ITS sequence heterogeneity poses challenges to ITS-based identification. Although D1/D2 sequencing offers an alternative, its utility may also be affected by intragenomic divergence in *R. microsporus*, highlighting the need for single-copy genes to improve species identification.

## INTRODUCTION

Members of the order Mucorales in the phylum Mucoromycota are ubiquitous, fast-growing filamentous fungi found in soil, decaying vegetation, and other organic substrates (1). Mucorales comprises several morphologically distinct families and genera, with some species being morphologically indistinguishable. Several thermotolerant species are clinically important opportunistic pathogens, including *Rhizopus microsporus*, *Rhizopus arrhizus*, and *Cunninghamella bertholletiae* (2). Accurate species identification is essential, as clinically relevant differences exist in epidemiology, pathogenicity, and antifungal susceptibility. For example, compared with *Cunninghamella echinulata* and *Cunninghamella elegans*, *C. bertholletiae* is more frequently associated with human disease, particularly pulmonary and disseminated mucormycosis, and is linked to higher mortality rates than other Mucorales species (3, 4). *R. microsporus* and *R. arrhizus* are common etiologic agents of rhino-orbital-cerebral, pulmonary, and gastrointestinal mucormycosis (2, 5). Notably, *R. microsporus* and *R. arrhizus* var. *arrhizus* are usually susceptible to the anti-Mucorales azoles posaconazole and isavuconazole, whereas *R. arrhizus* var. *delemar* exhibits reduced susceptibility to both agents (6).

The internal transcribed spacer (ITS) region of the ribosomal DNA, consisting of two non-coding sequences (ITS1 and ITS2) flanking the 5.8S rRNA gene, is widely used in fungal taxonomy and diagnostics. Estimates of ITS copy number vary substantially, with *in silico* read depth analyses suggesting 14–1,442 copies per fungal genome, whereas long-read sequencing indicates an average of about 15 copies (7, 8). Owing to its multicopy nature and clear barcode gap between inter- and intra-specific variation, the Fungal Working Group of the Consortium for the Barcode of Life identified the ITS region as the universal DNA barcode for fungi (9). For species identification of *Mucorales*, ITS sequencing is recommended in taxonomic studies and international guidelines as the primary tool (10, 11). However, the presence of multiple ITS copies within fungal genomes can complicate species-level identification. A genome-mining study reported intragenomic ITS variation in about two-thirds of fungal genomes with multiple ITS copies (65%, 419/641) and in nearly half of taxa (49.7%, 90/181) when only high-quality assemblies generated with both long- and short-read sequencing were analyzed (8). In Mucorales, Woo et al. demonstrated intragenomic ITS sequence heterogeneity in *R. microsporus* strains from Hong Kong by cloning and Sanger sequencing (12).

In our routine analyses, *C. bertholletiae* and *R. microsporus* isolates frequently showed mixed (double or multiple) peaks in chromatograms from conventional ITS Sanger sequencing, complicating sequence interpretation, whereas sequencing of the D1/D2 domain at the 5′ end of the 28S (LSU) rRNA gene sometimes enabled species identification. However, the sequence characteristics underlying this phenomenon in *C. bertholletiae* have not been described. To address this, this study investigated intragenomic ITS and D1/D2 variants in *C. bertholletiae*, *C. echinulata*, and *R. microsporus* by cloning and Sanger sequencing of the ITS-D1/D2 region and performed long-read whole-genome sequencing of one *C. bertholletiae* strain to clarify the molecular basis of ITS heterogeneity in these species.

## MATERIALS AND METHODS

### Fungal isolates

A total of 14 isolates collected in Taiwan were analyzed, including nine *C. bertholletiae* (seven clinical and two environmental) showing mixed ITS chromatograms by Sanger sequencing, three environmental *C. echinulata* selected at random, and two clinical *R. microsporus* (one showing mixed ITS chromatograms and the other showing scattered mixed peaks). All isolates were stored at −80°C in cryovials containing yeast malt broth and glycerol as haploid asexual vegetative hyphae with sporangiospores until analysis. For analysis, the isolates were subcultured and streaked onto Sabouraud dextrose agar (SDA) plates using a three-zone streaking method, and pure colonies were selected for further investigation. Species identification was initially performed by conventional sequencing of the ITS and D1/D2 regions using the primer pairs ITS1/ITS4 and NL1/NL4, respectively, as previously described (13, 14), with Taq DNA Polymerase 2× Master Mix RED (Ampliqon, Odense, Denmark) under the following PCR conditions: initial denaturation at 95°C for 5 min and 35 cycles of 98°C for 15 s, 60°C for 30 s, and 72°C for 40 s, followed by a final extension at 72°C for 5 min and a hold at 25°C. In addition, the ITS-D1/D2 region of *C. bertholletiae* NCK234 and *R. microsporus* NCK408 was sequenced using the ITS1/NL4 primer pair. Identification was further confirmed by sequencing the cloned ITS-D1/D2 amplicons, as described below. Detailed isolate and primer information and DDBJ/NCBI accession numbers for sequences generated in this study are summarized in Table 1.

**TABLE 1:** (A) Information on 14 Mucorales isolates analyzed. (B) PCR primers used in this study^*a*^

(A)						
Species	Strain no.	Year of recovery		Sources (crop)	ITS-D1/D2 sequences	DDBJ/NCBI accession no.
*Cunninghamella bertholletiae*	NCK083	2012		Ascites		
	NCK182	2013		Endotracheal aspirate	Cub-b-1-1 Cub-b-2-1 Cub-b-2-2	LC885645 LC885646 LC885647
	NCK230	2014		sputum		
	NCK234	2014		sputum	Cub-a-1-1 Cub-a-2-1 Cub-a-4-2	LC885638 LC885639 LC885644
	NCK716	2018		Pleural fluid	Cub-a-3-2 Cub-a-1-12 Cub-a-9-2 Cub-a-6-1 WGS	LC885640 LC885641 LC885642 LC885643 DRR724713
	NCK851	2019		Endotracheal aspirate		
	NCK867	2019		Pleural fluid		
	TD275.1	2015		Soils (turmeric)		
	PaS.17.4.1	2018		Soil (papaya)		
*Cunninghamella echinulata*	SD194Z1	2015		Soil (water spinach)	Cue-a-1-1	LC885648
	TC085.2	2015		Soil (farm)	Cue-b-1-1	LC885649
	SD127Z1	2015		Soil (chili pepper)	Cue-c-1	LC885650
*Rhizopus microsporus*	NCK408	2016		Endotracheal aspirate	Rpm-a-1-1 Rpm-c-1	LC885651 LC885653
	LCM385	2018		Sputum	Rpm-b-1	LC885652

(B)						
PCR primer		Target region	Sequence	Reference		
ITS1 (forward)		ITS1-5.8S-ITS2	5′-TCC GTA GGT GAA CCT GCG G-3′	(13)		
ITS4 (reverse)			5′-TCC TCC GCT TAT TGA TAT GC-3′	(13)		
NL1 (forward)		D1/D2 region	5′-GCA TAT CAA TAA GCG GAG GAA AAG-3′	(14)		
NL4 (reverse)			5′-GGT CCG TGT TTC AAG ACG G-3′	(14)		

^*a*^ WGS, whole genome sequence.

### ITS-D1/D2 PCR, cloning, and Sanger sequencing

Fungal isolates were cultured on SDA plates at 28°C for 48–72 h, and genomic DNA was subsequently extracted from the mycelia using the Quick-DNA Fungal/Bacterial Miniprep Kit (Zymo Research Corp., Irvine, CA, USA). For each isolate, an approximately 1,500 bp ITS-D1/D2 fragment encompassing the ITS1-5.8S-ITS2 and D1/D2 regions was amplified using the primer pair ITS1/NL4 and the above-mentioned DNA polymerase under the following PCR conditions: initial denaturation at 95°C for 5 min, 30 cycles of 98°C for 15 s, 60°C for 30 s, and 72°C for 80 s, followed by a final extension at 72°C for 5 min and a hold at 25°C. Amplified PCR products were resolved on 1.5% (wt/vol) agarose gels containing 5% EtB “Out” Nucleic Acid Staining Solution (Yeastern Biotech Co., Taipei, Taiwan) in Tris-borate-EDTA buffer at 100 V for 30 min and photographed under UV illumination. The products were then gel-purified with the QIAquick PCR Purification Kit (QIAGEN, Hilden, Germany), cloned into the pGEM-T vector (Promega Corp., Madison, WI), and transformed into *Escherichia coli* JM109 competent cells according to the manufacturer’s instructions. For each *C. bertholletiae* isolate, at least eight *E. coli* colonies were selected, and the cloned amplicons were sequenced on an ABI 3730XL DNA Analyzer (Applied Biosystems, Foster City, CA).

All cloned ITS-D1/D2 sequences, both within and between isolates, were aligned with MUSCLE v3.8.1551 implemented in SnapGene Viewer to assess intragenomic variation. Homopolymeric tracts, defined as stretches of more than four consecutive identical nucleotides, were examined in detail.

### ITS sequence analysis based on whole genome sequences

*C. bertholletiae* NCK716 was subjected to whole-genome sequencing. After retrieval from the −80°C freezer, the isolate was initially subcultured on potato dextrose agar until sporulation. Spores were then collected and incubated in Sabouraud dextrose broth at 37°C. After 16 h of incubation, genomic DNA was extracted from the resulting mycelia using the NucleoBond DNA extraction kit (Macherey-Nagel, Düren, Germany). DNA libraries were prepared according to the SMRTbell Express Template Prep Kit and HiFi Reads protocols, and sequencing was performed on the Sequel II System (PacBio, Menlo Park, CA) to generate long high-fidelity (HiFi) reads by circular consensus sequencing (CCS). Sequence reads were assembled into contigs using the SMRT Link v11.0.0 Microbial Assembly pipeline. To assess intragenomic ITS variation, raw reads mapping to the consensus ITS sequence were extracted and analyzed.

## RESULTS

### ITS and D1/D2 chromatograms from Sanger sequencing

**Fig 1:**
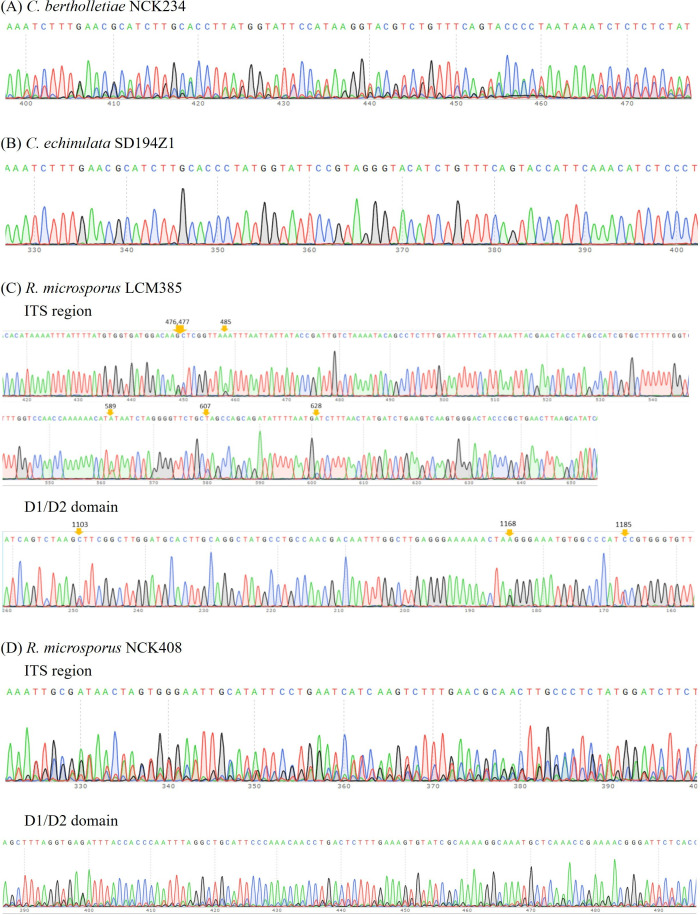
Sanger sequencing chromatograms of the ITS region from (**A**) *C. bertholletiae* NCK234, (**B**) *C. echinulata*, and (**C and D**) *R. microsporus*. Panels **C** and **D** also include D1/D2 chromatograms.

The extracted DNA from all isolates met the quality criteria, with A260/A280 and A260/A230 ratios of at least 1.8. Sanger sequencing chromatograms of the ITS and D1/D2 regions, generated using the ITS1/ITS4 and NL1/NL4 primer pairs, respectively, were inspected for each isolate (Fig. 1). For the ITS region, nine *C. bertholletiae* isolates exhibited extensive mixed peaks, whereas the three *C. echinulata* isolates produced predominantly clear chromatograms. Of the two *R. microsporus* isolates, NCK408 showed extensive mixed peaks, while LCM385 yielded an interpretable profile with only a few scattered double peaks. The D1/D2 chromatograms were generally interpretable for all isolates except NCK408, which also exhibited extensive mixed peaks. In addition, mixed chromatograms of the ITS region were detected in *C. bertholletiae* NCK234 and *R. microsporus* NCK408 during ITS-D1/D2 sequencing with the ITS1/NL4 primer pair (Fig. S1).

### Intragenomic ITS variation in *C. bertholletiae*

From nine *C. bertholletiae* isolates, 122 ITS-D1/D2 clones (8–28 per isolate) were obtained by cloning and sequenced by Sanger sequencing. Alignment of all clones revealed six homopolymeric tracts, which show subtle length variations within the ITS1 and ITS2 regions, designated S1–S6 (Table 2). Based on homopolymer lengths, the clones were assigned to 31 ITS sequence types, which were classified into two groups: Cub-a in six isolates (types Cub-a-1 to Cub-a-20) and Cub-b in three isolates (Cub-b-1 to Cub-b-11), with Cub-b characterized by nearly identical homopolymer lengths at S3 and S4. Variants containing additional single-nucleotide polymorphisms (SNPs) within the ITS-D1/D2 regions were designated with an additional suffix number (e.g., Cub-a-1-1 within Cub-a-1).

**TABLE 2:** Intragenomic ITS sequence variation among nine *C. bertholletiae* isolates^*a*^

Strain	ITS sequence type	No. (%) of clones	Nucleotide positions of homopolymeric tracts (bp)					
			ITS1			ITS2		
			65–73 (S1)	210–218 (S2)	329–337 (S3)	521–529 (S4)	560–572 (S5)	613–620 (S6)
NCK234	**Cub-a-1**	**4 (40**)	(**T)9**	(**G)9**	(**A)9**	(**A)9**	(**T)13**	(**T)8**
(10 clones, five types)	Cub-a-2	1 (10)					(T)12	
	Cub-a-3	1 (10)					(T)14	
	Cub-a-4	3 (30)		(G)8				
	Cub-a-5	1 (10)	(T)8				(T)12	
NCK230	Cub-a-1	6 (66.7)						
(nine clones, four types)	Cub-a-2	1 (11.1)					(T)12	
	Cub-a-6	1 (11.1)	(T)8					
	Cub-a-7	1 (11.1)		(G)10				
NCK716	Cub-a-1	10 (40)						
(25 clones, 10 types)	Cub-a-2	5 (20)					(T)12	
	Cub-a-3	1 (4)					(T)14	
	Cub-a-6	2 (8)	(T)8					
	Cub-a-8	1 (4)						(T)7
	Cub-a-9	2 (8)			(A)8		(T)12	
	Cub-a-10	1 (4)			(A)8		(T)14	
	Cub-a-11	1 (4)			(A)10			
	Cub-a-12	1 (4)	(T)8		(A)8			
	Cub-a-13	1 (4)	(T)8				(T)11	
NCK851	Cub-a-1	8 (66.8)						
(12 clones, five types)	Cub-a-2	1 (8.3)					(T)12	
	Cub-a-14	1 (8.3)				(A)8		
	Cub-a-15	1 (8.3)		(G)8	(A)8		(T)12	(T)7
	Cub-a-16	1 (8.3)		(G)10		(A)10		
NCK867	Cub-a-1	3 (37.5)						
(eight clones, five types)	Cub-a-3	2 (25)					(T)14	
	Cub-a-5	1 (12.5)	(T)8				(T)12	
	Cub-a-9	1 (12.5)			(A)8		(T)12	
	Cub-a-17	1 (12.5)			(A)8			
TD275.1	Cub-a-1	4 (40)						
(10 clones, six types)	Cub-a-2	2 (20)					(T)12	
	Cub-a-3	1 (10)					(T)14	
	Cub-a-18	1 (10)					(T)11	
	Cub-a-19	1 (10)				(A)10		
	Cub-a-20	1 (10)	(T)10	(G)8			(T)14	
NCK182	Cub-b-1	6 (60)			(A)7	(A)8		
(10 clones, four types)	Cub-b-2	2 (20)			(A)7	(A)8	(T)12	
	Cub-b-3	1 (10)			(A)7	(A)8		(T)7
	Cub-b-4	1 (10)	(T)8		(A)7	(A)8	(T)11	
NCK083	Cub-b-1	17 (60.7)			(A)7	(A)8		
(28 clones, six types)	Cub-b-2	5 (17.9)			(A)7	(A)8	(T)12	
	Cub-b-5	1 (3.6)			(A)7	(A)8	(T)12	C(T)7
	Cub-b-6	1 (3.6)			(A)7	(A)8	(T)14	
	Cub-b-7	2 (7.1)	(T)8		(A)7	(A)8		
	Cub-b-9	2 (7.1)	(T)10		(A)7	(A)8		
PaS.17.4.1	Cub-b-1	5 (50)			(A)7	(A)8		
(10 clones, six types)	Cub-b-2	1 (10)			(A)7	(A)8	(T)12	
	Cub-b-6	1 (10)			(A)7	(A)8	(T)14	
	Cub-b-8	1 (10)	(T)8		(A)7	(A)7G		
	Cub-b-10	1 (10)	(T)8		(A)7	(A)8	(T)12	
	Cub-b-11	1 (10)		(G)10	(A)7	(A)8		

^*a*^ Cub-a-1-1 of the Cub-a-1 sequence type was used as the reference. Variants are denoted by a capital letter indicating the homopolymer nucleotide (A, adenine; T, thymine; and G, guanine), followed by the number of consecutive bases. Homopolymer lengths identical to the reference are left blank.

**Fig 2:**
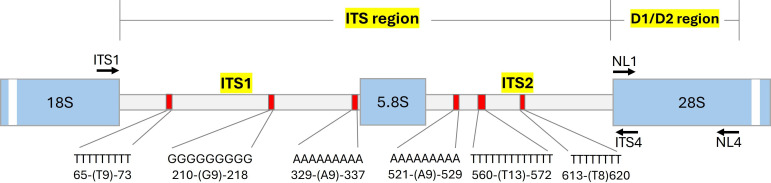
ITS and D1/D2 regions of the rRNA gene cluster from *C. bertholletiae* NCK234, showing six homopolymeric tracts (S1–S6, red bars) with nucleotide positions, the homopolymer base (A, T, and G), the number of repeats in parentheses, and primer positions indicated by arrows.

The predominant ITS-D1/D2 sequence from NCK234 (Cub-a-1-1) was used as the reference (Fig. 2), with homopolymer nucleotides and lengths (bp) at S1–S6 as follows (ranges from other isolates in parentheses): T9 (8–10), G9 (8–10), A9 (7–10), A9 (7–10), T13 (11–14), and T8 (7, 8). Cub-a-1 and Cub-b-1, which shared 99.45% ITS sequence identity, were the most common ITS sequence types in their respective groups, representing ≥38% and ≥50% of clones in each isolate. A two-nucleotide length difference at one or more of the six homopolymeric tracts was observed in all but one isolate (NCK230). Ten additional homopolymeric tracts (4–7 bp) were identified in the ITS-D1/D2 regions of all isolates; none showed length variation (Table S1). Overall, each *C. bertholletiae* isolate contained at least four ITS sequence types based on homopolymer lengths. In addition, few SNPs were identified in the ITS-D1/D2 of certain isolates, with 1–3 SNPs in the ITS of nine isolates and 1–2 SNPs in the D1/D2 of eight isolates (Table S2). Among nine isolates, intragenomic sequence divergence ranged from 0.14% to 0.55% in ITS and from 0% to 0.41% in D1/D2 (Table 3). No intragenomic sequence variation was detected in the 5.8S rRNA gene.

**TABLE 3:** Intragenomic divergence in the ITS and D1/D2 regions by cloning and Sanger sequencing

	Sequence divergence %	
Strain	ITS region	D1/D2 domain
*C. bertholletiae*		
NCK234	0.27%	0.27%
NCK230	0.14%	0.00%
NCK716	0.41%	0.27%
NCK851	0.55%	0.27%
NCK867	0.27%	0.14%
TD275.1	0.55%	0.41%
NCK182	0.41%.	0.14%
NCK083	0.41%	0.27%
PaS.17.4.1	0.41%	0.14%
*C. echinulata*		
SD194Z1	0.23%	0.27%
TC085.2	0.00%	0.41%
SD127Z1	0.00%	0.00%
*R. microsporus*		
LCM385	1.19%	1.43%
NCK408	27.37%	3.10%

For PacBio HiFi whole-genome sequencing of *C. bertholletiae* NCK716, 1,238 raw reads mapping to the ITS region were analyzed. The distribution of reads containing different homopolymer lengths at S1–S6 is shown in Table 4. PacBio sequencing results were consistent with cloning and Sanger sequencing in both the predominant homopolymeric lengths observed at S1–S6 and the relative proportions of variants. For example, at S5, cloning and Sanger sequencing yielded T-homopolymer lengths of 13 bp (60.0%), 12 bp (28.0%), 14 bp (8.0%), and 11 bp (4.0%) among 25 clones, closely matching PacBio reads: 13 bp (62.5%), 12 bp (32.6%), 14 bp (4.7%), and 11 bp (0.2%) among 1,238 reads.

**TABLE 4:** Proportions of different homopolymer lengths at six tracts (S1–S6) in *C. bertholletiae* NCK716, based on 25 clones obtained by cloning-Sanger sequencing and 1,238 ITS-mapped reads from PacBio HiFi whole-genome sequencing^*a*^

	Homopolymeric tract variants (%) by nucleotide position					
Method	65–73 (S1)	210–218 (S2)	329–337 (S3)	521–529 (S4)	560–572 (S5)	613–620 (S6)
Cloning-Sanger	T (9): 84.0% T (8): 16.0%	G (9): 100%	(A)9: 80.0% (A)8: 16.0% (A)10: 4.0%	(A)9: 100%	(T)13: 60.0% (T)12: 28.0% (T)14: 8.0% (T)11: 4.0%	(T)8: 96.0% (T)7: 4.0%
PacBio HiFi	(T)9: 87.3% (T)8: 11.9% A(T)7: 0.1% A(T)8: 0.1% (T)10: 0.6%	(G)9: 95.7% (G)8: 3.0% (G)10: 1.0% (G)7: 0.1% C(G)10: 0.1% (G)11: 0.2%	(A)9: 85.3% (A)8: 6.7% (A)10: 7.9% C(A)9: 0.1%	(A)9: 98.5% (A)8: 0.9% (A)11: 0.6%	(T)13: 62.5% (T)12: 32.6% (T)14: 4.7% (T)11: 0.2%	(T)8: 96.6% (T)7: 3.2% C(T)8: 0.1% (T)9: 0.2%

^*a*^ Variants are denoted by the homopolymer nucleotide (capital letter) followed by the number of consecutive repeats.

Cub-a-1-1 and Cub-b-1-1 showed 99.20% and 99.33% ITS sequence identity, respectively, to *C. bertholletiae* ATCC 42215, and 99.86% identity each for the D1/D2 domain of the same strain (GenBank accession no. FJ345351.1).

### Intragenomic ITS variation in *C. echinulata*

Analysis of 10–11 ITS-D1/D2 clones from each of three *C. echinulata* isolates revealed strain-specific ITS patterns: Cue-a (SD194Z1), Cue-b (TC085.2), and Cue-c (SD127Z1) (Table 5). Intragenomic ITS variation was observed only in SD194Z1, which contained five ITS sequence types differing by 1–2 SNPs, with Cue-a-1-1 as the predominant type. Fourteen homopolymeric tracts (13 of 5–7 bp and one of 8 bp) were identified in the ITS-D1/D2 regions of all isolates; none showed length variation (Table S1). Minimal intragenomic variation (≤3 SNPs) was detected in the D1/D2. Among the three isolates, intragenomic sequence divergence ranged from 0% to 0.23% in the ITS and from 0% to 0.41% in the D1/D2. A single SNP was identified in the 5.8S in one clone of SD194Z1.

**TABLE 5:** Intragenomic ITS and D1/D2 sequence variation among three *C. echinulata* isolates^*a*^

Strain	ITS sequence type	No. (%) of clones	Nucleotide positions (bp)																															
			ITS1											5.8S	ITS2												D1/D2 domain							
			53^ 54	62	91	122	123	126	128	138	147	152	170	408	447	500	573	614	625	775	794-797	802-805	807-809	812-813	820-822	826	1084	1104	1173	1277	1325	1430	1443	1600
SD194Z1 (11 clones, five types)	**Cue-a-1-1**	**6 (55)**		**T**	**G**	**A**	**G**	**T**	**A**	**T**	**A**	**T**	**A**	**T**	**A**	**T**	**T**	**T**	**T**	**A**	**GC** **TC**	**GTTC**	**TTA**	**AA**	**TGC**	**C**	**G**	**A**	**G**	**T**	**T**	**G**	**A**	**A**
	Cue-a-1-2	1 (9)																													C		G	
	Cue-a-2	1 (9)											G																					
	Cue-a-3	1 (9)														C																		G
	Cue-a-4	1 (9)				G																												
	Cue-a-5	1 (9)												C						G							A							
TC085.2 (10 clones, one type)	Cue-b-1-1	9 (90)	TAA														C		C													T		
	Cue-b-1-2	1 (10)	TAA														C		C										A	C				
SD127Z1 (10 clones, one type)	Cue-c-1	10 (100)		–	A		A	G	T	–	T	–			–		C	C			AA GT	AC TT	–	GG	C-T	T		G						

^*a*^ Cue-a-1-1 was used as the reference. SNPs at the indicated nucleotide positions are shown, with positions identical to the reference left blank. Capital letters denote nucleotides. The column “53^54” indicates the insertion site between nucleotide positions 53 and 54. “-” indicates the absence of a nucleotide at the indicated position.

Cue-a-1, Cue-b-1, and Cue-c-1 showed 99.65%, 99.53%, and 97.17% ITS sequence identity, respectively, to *C. echinulata* CBS 766.68 (JN205894.1), and 98.38%, 98.25%, and 98.25% for the D1/D2 domain of the same strain (MH877699.1).

### Intragenomic ITS variation in *R. microsporus*

**Fig 3:**
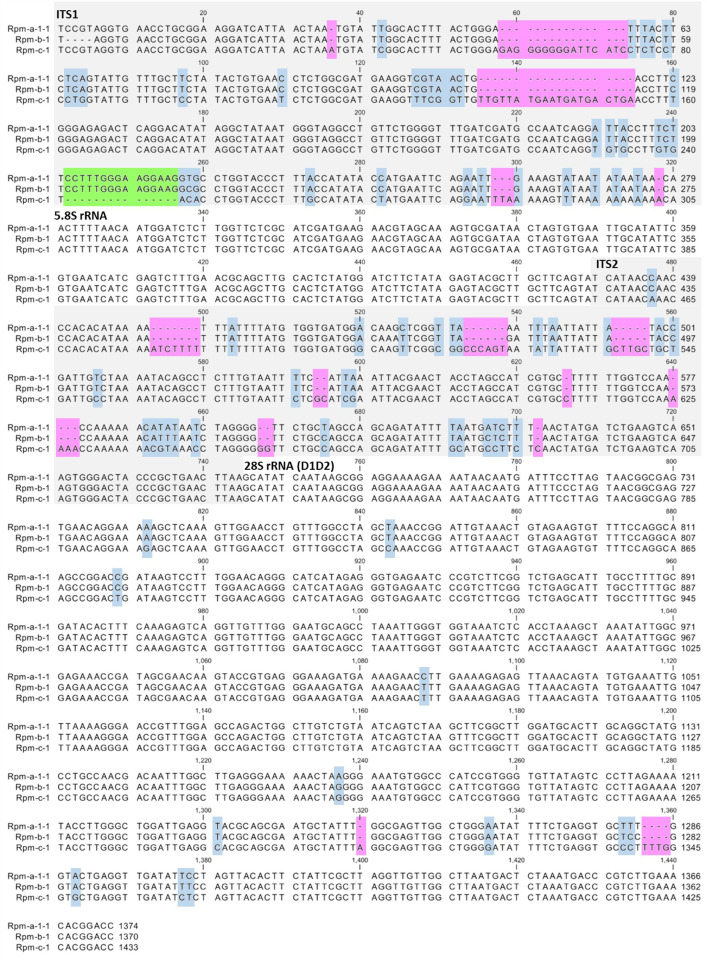
Alignment of three ITS and D1/D2 sequence types from *R. microsporus* strains LCM385 and NCK408. The ITS1 and ITS2 regions are indicated in gray. Insertions and deletions (indels) are highlighted in pink and green, and SNPs are shown in blue.

Analysis of 9–10 ITS-D1/D2 clones from each of the two *R. microsporus* isolates revealed 12 distinct ITS sequence types, which were classified into three groups: Rpm-a, Rpm-b, and Rpm-c (Table 6). LCM385 harbored two divergent groups, Rpm-a (Rpm-a-1 to Rpm-a-3) and Rpm-b (Rpm-b-1 to Rpm-b-3), with 99.86% sequence identity between the reference sequences Rpm-a-1-1 and Rpm-b-1. Groups Rpm-a and Rpm-b differed by seven fixed SNPs, with an additional 0–1 SNP among clones. NCK408 also contained two divergent groups, Rpm-a (Rpm-a-1 and Rpm-a-4) and Rpm-c (Rpm-c-1 to Rpm-c-6), which showed substantial differences. Compared with Rpm-a-1-1, Rpm-c-1 had 13 insertions (1–20 bp each) and a 15-bp deletion in the ITS, resulting in a 59-bp length difference and only 72.99% sequence identity (Fig. 3). ITS sequence types belonging to the Rpm-c group differed by 0–3 SNPs and exhibited length variation at two homopolymeric tracts (6–8 bp and 11–12 bp). Four additional homopolymeric tracts (5–7 bp) were identified in the ITS2-D1/D2 regions of both isolates, all without length variation (Table S1).

**TABLE 6:** Intragenomic ITS and D1/D2 variation among two *R. microsporus* isolates. Rpm-a-1-1 was used as the reference for LCM385, and Rpm-a-1-1 together with Rpm-c-1 were used as the references for NCK408^*a*^

Strain	ITS sequence type	No. (%) of clones	Nucleotide positions (bp)																						
			ITS1				5.8S	ITS2					D1/D2 domain												
			47	62	166	221	379	476-477	485	589	607	628	679	697	718	868	923	987	1019	1103	1168	1185	1284-1285	1304	1306
LCM385 (nine clones, six types)	**Rpm-a-1-1**	**1**	**T**	**T**	**T**	**T**	**A**	**GC**	**A**	**A**	**T**	**A**	**T**	**A**	**T**	**T**	**A**	**A**	**C**	**C**	**A**	**C**	**TT**	**C**	**T**
	Rpm-a-1-2	1																G	T						
	Rpm-a-2	1	C										C				G		T						
	Rpm-a-3	1			C														T						
	Rpm-b-1	3				C		AT	G	T	C	C			C				T	T	G	T	CC	T	C
	Rpm-b-2	1				C	G	AT	G	T	C	C		G	C				T	T	G	T	CC	T	C
	Rpm-b-3	1		C		C		AT	G	T	C	C			C				T	T	G	T	CC	T	C
			47	62	166	221	379	476-477	485	589	607	628	679	697	718	868	923	987	1019	1103	1168	1185	1284-1285	1304	1306
NCK408 (10 clones, seven types)	**Rpm-a-1-1**	**1**	**T**	**T**	**T**	**T**	**A**	**GC**	**A**	**A**	**T**	**A**	**T**	**A**	**T**	**T**	**A**	**A**	**C**	**C**	**A**	**C**	**TT**	**C**	**T**
	Rpm-a-1-3	1														C			T						
			293-299					481-494	617	651	696	714-715	824	1317											
	**Rpm-c-1**	**2**	(**A)7**					(**T)12AT**	**T**	**G**	**T**	**TA**	**T**	**T**											
	Rpm-c-2	1										CA	C												
	Rpm-c-3	1							C	A															
	Rpm-c-4	1						(T)11AC			C	TG													
	Rpm-c-5	1	(A)8					(T)11AT																	
	Rpm-c-6-1	1	(A)6																						
	Rpm-c-6-2	1	(A)6											C											

^*a*^ SNPs at the indicated nucleotide positions are shown, with positions identical to the reference left blank. Capital letters denote nucleotides. Compared with Rpm-a-1-1, Rpm-c-1 exhibited additional indels in the ITS and 12 SNPs plus a 4-bp insertion in the D1/D2 region (Fig. 3).

LCM385 contained two divergent D1/D2 sequence groups corresponding to its two ITS sequence groups. They differed by eight fixed SNPs, consistent with the double peaks observed at the corresponding positions in the chromatogram (Fig. 1C). NCK408 also harbored two divergent D1/D2 sequence groups. Compared with Rpm-a-1-1, Rpm-c-1 contained 12 SNPs and two insertions (1–4 bp) in D1/D2, resulting in a mixed chromatogram. Overall, the intragenomic divergence in the ITS and D1/D2 regions was 1.19% and 1.43%, respectively, in LCM385, and 27.37% and 3.10%, respectively, in NCK408. A single SNP was identified in the 5.8S in one clone of LCM385.

**Fig 4:**
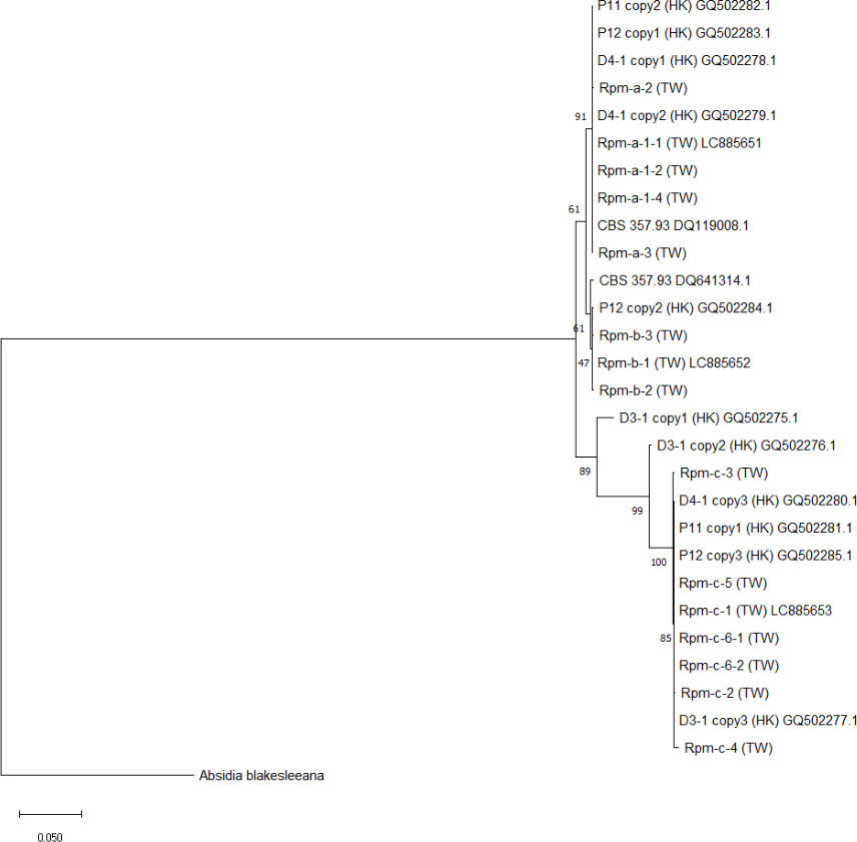
Neighbor-joining phylogenetic tree based on ITS sequences derived from two *Rhizopus microsporus* isolates from Taiwan (TW) in this study, the Hong Kong (HK) isolates from patients (P11 and P12) and allopurinol tablets (D3-1 and D4-1) (12), and strain CBS 357.93. Accession numbers are indicated. The tree was generated using Jukes-Cantor correction with 1,000 bootstrap replicates.

The intragenomic hybrid ITS sequence patterns identified herein corresponded closely to *R. microsporus* sequences from patients (P11 and P12) and allopurinol tablets (D3-1 and D4-1) in Hong Kong (12). Strain P11 harbored copy 2 (GQ502279.1) with 99.41% identity to Rpm-a-1-1 and copy 1 (GQ502281.1) with 99.86% identity to Rpm-c-1-1. Strain D4-1 harbored copy 2 (GQ502279.1) with 100% identity to Rpm-a-1-1 and copy 3 (GQ502280.1) with 99.86% identity to Rpm-c-1-1. Strain P12 harbored copy 1 (GQ502283.1) with 99.41% identity to Rpm-a-1-1, copy 2 (GQ502284.1) with 99.85% identity to Rpm-b-1, and copy 3 (GQ502285.1) with 99.86% identity to Rpm-c-1-1. Strain D3-1 harbored copy 3 (GQ502277.1) with 100% identity to Rpm-c-1-1. The phylogenetic relationships among ITS sequences obtained from the *R. microsporus* isolates in this study and those reported from Hong Kong are shown in Fig. 4.

## DISCUSSION

The ITS regions of rDNA are widely used in fungal taxonomy because they are easily amplified and sufficiently variable for species-level identification (9). As members of a multigene family, rDNA repeats usually undergo concerted evolution, minimizing intragenomic variation while maintaining divergence between species (15). Nonetheless, intragenomic variation, defined as sequence differences among multiple copies within a genome, may arise from either incomplete homogenization or hybridization between divergent lineages (15, 16). Compared with the coding regions of rDNA (SSU, 5.8S, and LSU), which are more conserved under selective pressure to maintain function, the non-coding ITS1 and ITS2 regions are more tolerant of mutational events (insertions, deletions, and replication slippage), leading to intragenomic diversity (15). Organisms within early-diverging taxa, including Mucoromycota, Chytridiomycota, and Zoopagomycota, tend to exhibit the highest levels of intragenomic variation (8). While reports on ITS variation in *Mucorales* are limited, this study provides the first evidence of intragenomic ITS heterogeneity in *C. bertholletiae*, revealed by cloning with Sanger sequencing as well as PacBio long-read genome analysis. We also confirmed previously reported hybrid ITS sequence patterns in *R. microsporus* (12), underscoring that such variation is a prevalent feature.

*C. bertholletiae* predominantly exhibits an asexual life cycle in the environment, with haploid mycelia developing from asexual spores, whereas zygospore formation is rarely observed (17, 18). Laboratory identification therefore relies mainly on asexual structures, characterized by single-spored sporangiola, and on the absence of zygospore formation, which serves as a diagnostic character in identification keys (18, 19). The mortality of mucormycosis caused by *C. bertholletiae* is high, reflecting both its inherent virulence and reduced susceptibility to amphotericin B (3, 20), underscoring the need for accurate species-level identification.

In this study, length variation at six homopolymeric tracts ≥ 8 bp in the ITS region was identified as the primary cause of mixed peaks in Sanger chromatograms in *C. bertholletiae*. To date, no studies have specifically reported homopolymeric tracts within the ITS of Mucorales. The observed length variation may partly result from polymerase slippage, a replication or sequencing error that occurs when DNA polymerase dissociates from the template while copying repetitive sequences, particularly homopolymeric tracts; misalignment upon reattachment can then lead to nucleotide insertions or deletions (21, 22). In *C. bertholletiae*, length variation in ITS homopolymeric tracts may result from genuine intragenomic differences or artifacts from Sanger sequencing of long homopolymers. Biologically, homopolymeric tracts are particularly prone to polymerase slippage, resulting in heritable length variation during asexual DNA replication. Technically, although Sanger sequencing is generally accurate, its reliability decreases with increasing homopolymer length. Tracts longer than 8–9 bases frequently generate *n* ± 1 peaks and downstream frameshifts, as noted in instrument manufacturers’ technical guidance (23). This is consistent with our finding that length variation in *C. bertholletiae* occurred mostly at homopolymeric tracts 8-14 bp in length, whereas tracts shorter than 8 bp rarely showed variation. Although it was not possible to definitively determine whether the observed length variations represent genuine biological differences or sequencing artifacts, genuine variation remains likely, as two-nucleotide length differences at homopolymeric tracts could be identified in most *C. bertholletiae* isolates. Furthermore, optimization of CCS has improved the accuracy of PacBio sequencing, generating highly accurate HiFi reads and reducing sequencing errors (24). The comparable proportions of length variants identified by cloning-Sanger sequencing and PacBio HiFi genome mining support that the variation is biological in origin. Notably, aside from homopolymer length variation, intragenomic ITS copies in *C. bertholletiae* were largely homogenized, with none or only a few SNPs detected. This pattern may be attributed to its predominantly asexual life cycle in the environment, which limits opportunities for genetic exchange with other strains.

Like *C. bertholletiae*, *C. echinulata* produces single-spored sporangiola and predominantly exhibits an asexual life cycle; the absence of zygospore formation serves as a diagnostic character in identification keys (17, 18, 25). *C. echinulata* shows low intragenomic ITS sequence diversity, with none or only minimal divergence (≤ 2 SNPs), which may be attributed to the absence of long homopolymeric tracts and a predominantly asexual life cycle. This limited variation results in double peaks only at polymorphic sites, while still yielding interpretable Sanger chromatograms. These findings suggest that *C. echinulata* can be more readily identified by molecular methods, although its role in human disease appears limited, with only a few infections reported (26, 27).

*R. microsporus* is heterothallic and produces zygospores when opposite mating types interact under appropriate conditions (28). In one study involving 48 *R. microsporus* isolates, 40 (83.3%) produced zygospores when mated with tester strains (28). In our study, both *R. microsporus* strains harbored hybrid ITS sequence patterns. In LCM385, SNPs between the two groups resulted in 1.19% intragenomic divergence, whereas in NCK408, substantial indels between the two groups led to 27.37% divergence, far exceeding interspecific variation. In the latter strain, the 59-bp size difference did not yield a thick band on the electrophoresis gel, but an indel beginning at the proximal position caused downstream frameshift, producing unreadable chromatograms. Notably, *R. microsporus* strains in this study shared hybrid ITS patterns similar to those of strains from Hong Kong (12). An earlier study reported two divergent ITS types within the *Fusarium* genome, hypothesized to result from either duplication before speciation or ancient hybridization (16). Likewise, studies in Hong Kong and ours revealed a comparable scenario in *R. microsporus*, supporting the hypothesis of ancient hybridization between divergent lineages that gave rise to hybrid ITS patterns, stably inherited and regionally disseminated.

In conventional PCR-Sanger sequencing, laboratory indicators of intragenomic ITS heterogeneity include the following: (i) thick electrophoresis bands in isolates with substantial ITS indels, causing PCR product size polymorphisms; (ii) mixed peaks at SNP positions in chromatograms, with base calling still feasible when only a few SNPs are present; and (iii) extensive chromatogram ambiguity in isolates with homopolymer length variations (as in *C. bertholletiae*) or indels (as in *R. microsporus*), leading to downstream frameshifts. In such situations, cloning of PCR products or high-throughput long-read sequencing is required to recover the intragenomic ITS variants. An alternative for species identification is sequencing the D1/D2 domain, which is feasible in isolates lacking long homopolymeric tracts or indels in this region, such as *C. bertholletiae*, *C. echinulata*, and *R. microsporus* LCM385. However, as seen in *R. microsporus* NCK408, this approach may be problematic in isolates with hybrid D1/D2 sequence patterns, particularly when indels produce unreadable chromatograms or when intragenomic divergence exceeds interspecific variation (e.g., 3.10% in NCK408), complicating taxonomic delimitation. In these instances, alternative single-copy genes such as RNA polymerase II subunits (RPB1 and RPB2), translation elongation factor 1-alpha (TEF1α), and beta-tubulin (TUB2), individually or in combination, may be explored for improved species identification and taxonomic delimitation (8).

While contamination or DNA degradation cannot be completely excluded as potential contributors to minor sequence variation, the reproducible species-specific and strain-specific chromatogram patterns, cross-method consistency, and acceptable DNA purity after extraction suggest that the observed ITS heterogeneity most likely reflects genuine intragenomic variation. This study is limited by the relatively small number of isolates included, and further comparison with publicly available sequences remains constrained because most genomic and ITS data sets are available only as consensus sequences or short Illumina reads that do not permit direct assessment of intragenomic ITS heterogeneity. Nevertheless, in addition to the Hong Kong isolates, we identified one *R. microsporus* var. *azygosporus* CBS 357.93, harboring two ITS sequence types similar to the Rpm-a and Rpm-b groups observed in our isolates, supporting a hybrid ITS pattern (Fig. 4). For *C. bertholletiae*, ITS sequences deposited in NCBI showed length variation at six homopolymeric tracts among strains (Fig. S2), indirectly supporting the intragenomic homopolymer length variation observed in our isolates.

In conclusion, our study reveals patterns of ITS heterogeneity, including length variation in long homopolymeric tracts in *C. bertholletiae* and hybrid ITS patterns in *R. microsporus*, that contribute to Sanger chromatogram ambiguity and pose challenges in ITS-based identification. Although D1/D2 sequencing offers an alternative, its utility may also be affected by intragenomic divergence in *R. microsporus*, highlighting the need for single-copy genes to improve species identification.

## Data Availability

The sequences generated in this study have been deposited in the DDBJ database, and the corresponding DDBJ/NCBI accession numbers are listed in Table 1.

## SUPPLEMENTAL MATERIAL

Tables S1 and S2; Figures S1 and S2 spectrum.03485-25-s0001.docx Table S1: Homopolymeric tracts in ITS-D1/D2. Table S2: Counts and percentages of ITS and D1/D2 clones with SNPs. Figure S1: Sanger sequencing chromatograms of the ITS region using the ITS1/NL4 primer pair. Figure S2: Multiple sequence alignment of the ITS region of *C. bertholletiae* isolates.

ASM does not own the copyrights to Supplemental Material that may be linked to, or accessed through, an article. The authors have granted ASM a non-exclusive, world-wide license to publish the Supplemental Material files. Please contact the corresponding author directly for reuse.
