# Supplementary material for: Challenges in ITS-based identification of Cunninghamella bertholletiae and Rhizopus microsporus due to intragenomic ITS sequence heterogeneity
Source: Microbiol Spectr. 2026 Jul 6;14(8):e03485-25. doi: 10.1128/spectrum.03485-25 (PMC13436413; doi:10.1128/spectrum.03485-25)
Supplement: Supplementary file 1 [file spectrum.03485-25-s0001.docx]

**Supplementary data**

**Table S1.** Homopolymeric tracts >4 bp in the ITS and D1/D2 regions of (A) nine *C. bertholletiae* (Cub-a-1-1 as reference), (B) three *C. echinulata* (Cue-a-1-1 as reference), and (C) two *R. microsporus* (Rpm-a-1-1) isolates. Each tract is denoted by a capital letter for the nucleotide followed by the number of consecutive bases. No homopolymeric tract length variation was observed among clones within each isolate.

| (A) | *C. bertholletiae* \| no. of clones \| Nucleotide positions (bp) \| \| \| \| \| \| \| \| \| \| \|; \| \| ITS1 \| \| \| \| 5.8S \| ITS2 \| \| \| \| D1/D2 \| \|; \| \| 55-59 \| 79-84 \| 204-208 \| 310-315 \| \| 576-580 \| 640-644 \| 664-668 \| 700-705 \| 1113-1117 \| 1238-1244 \|; NCK234 \| 10 \| (A)5 \| (A)6 \| (T)5 \| (T)6 \| - \| (A)5 \| (A)5 \| (T)5 \| (T)6 \| (A)5 \| (A)7 \|; NCK230 \| 9 \| (A)5 \| (A)6 \| (T)5 \| (T)6 \| - \| (A)5 \| (A)5 \| (T)5 \| (T)6 \| (A)5 \| (A)7 \|; NCK716 \| 25 \| (A)5 \| (A)6 \| (T)5 \| (T)6 \| - \| (A)5 \| (A)5 \| (T)5 \| (T)6 \| (A)5 \| (A)7 \|; NCK851 \| 12 \| (A)5 \| (A)6 \| (T)5 \| (T)6 \| - \| (A)5 \| (A)5 \| (T)5 \| (T)6 \| (A)5 \| (A)7 \|; NCK867 \| 8 \| (A)5 \| (A)6 \| (T)5 \| (T)6 \| - \| (A)5 \| (A)5 \| (T)5 \| (T)6 \| (A)5 \| (A)7 \|; TD275.1 \| 10 \| (A)5 \| (A)6 \| (T)5 \| (T)6 \| - \| (A)5 \| (A)5 \| (T)5 \| (T)6 \| (A)5 \| (A)7 \|; NCK182 \| 10 \| (A)5 \| (A)6 \| (T)5 \| (T)6 \| - \| (A)5 \| (A)5 \| (T)5 \| (T)6 \| (A)5 \| (A)7 \|; NCK083 \| 28 \| (A)5 \| (A)6 \| (T)5 \| (T)6 \| - \| (A)5 \| (A)5 \| (T)5 \| (T)6 \| (A)5 \| (A)7 \|; PaS.17.4.1 \| 10 \| (A)5 \| (A)6 \| (T)5 \| (T)6 \| - \| (A)5 \| (A)5 \| (T)5 \| (T)6 \| (A)5:9 clone AAGAA:1 clone \| (A)7 \| |
| --- | --- |
| (B) | *C. echinulata* \| no. of clones \| Nucleotide positions (bp) \| \| \| \| \| \| \| \| \| \| \| \| \| \| \|; \| \| ITS1 \| \| \| \| \| \| 5.8S \| ITS2 \| \| \| \| \| D1/D2 \| \| \|; \| \| 62-66 \| 112- 119 \| 162- 172 \| 185- 191 \| 240- 244 \| 260-264 \| \| 440- 446 \| 713- 718 \| 745- 749 \| 787- 793 \| 834- 840 \| 997- 1001 \| 1238- 1242 \| 1364- 1369 \|; SD194Z1 \| 11 \| (T)5 \| (T)8 \| (A)6 \| (T)7 \| (A)5 \| (A)5 \| - \| (T)7 \| (A)6 \| (T)5 \| (A)7 \| (T)7 \| (T)5 \| (A)5 \| (A)6 \|; TC085.2 \| 10 \| (T)5 \| (T)8 \| (A)6 \| (T)7 \| (A)5 \| (A)5 \| - \| (T)7 \| (A)6 \| (T)5 \| (A)7 \| (T)7 \| (T)5 \| (A)5 \| (A)6 \|; SD127Z1 \| 10 \| (T)4 \| (T)8 \| (A)6 \| (T)7 \| (A)5 \| (A)5 \| - \| (T)7 \| (A)6 \| (T)5 \| (A)7 \| (T)7 \| (T)5 \| (A)5 \| (A)6 \| |
| (C) | *R. microsporus*, ITS seqeunce type \| no. of clones \| Nucleotide positions (bp) \| \| \| \| \|; \| \| ITS \| 5.8S \| ITS2 \| \| D1/D2 \|; \| \| \| \| 565-570 \| 580-585 \| 740-744 \| 1159-1164; NCK408, Rpm-a \| 10 \| - \| - \| (T)6 \| (A)6 \| (A)5 \| (A)6; LCM385, Rpm-a and Rpm-b \| 9 \| - \| - \| (T)6 \| (A)6 \| (A)5 \| (A)6 |

**Table S2.** Counts and percentages of ITS and D1/D2 clones with SNPs relative to the reference sequence Cub-a-1-1 in nine *C. bertholletiae* isolates.

| Strain | No. of clones | ITS, no. of clones (%) | | | | D1/D2, no. of clones (%) | |
| --- | --- | --- | --- | --- | --- | --- | --- |
|  |  | 1 SNP | | 2 SNPs | 3 SNPs | 1 SNP | 2 SNPs |
| NCK083 | 28 | | 5 (17.8) | 3 (10.7) | 0 | 6 (21.4) | 2 (7.1) |
| NCK182 | 10 | | 0 | 0 | 1 (10) | 3 (30) | 0 |
| NCK230 | 9 | | 1 (11) | 0 | 0 | 0 | 0 |
| NCK234 | 10 | | 2 (20) | 0 | 0 | 3 (30) | 1 (10) |
| NCK716 | 25 | | 6 (24) | 0 | 0 | 6 (24) | 1 (4) |
| NCK851 | 12 | | 2 (16.7) | 0 | 0 | 2 (16.7) | 1 (8.3) |
| NCK867 | 8 | | 2 (25) | 0 | 0 | 1 (12.5) | 0 |
| PaS.17.4.1 | 10 | | 3 (30) | 0 | 0 | 3 (30) | 0 |
| TD275.1 | 10 | | 4 (40) | 2 (20) | 0 | 4 (40) | 2 (20) |

**Fig. S1.** Sanger sequencing chromatograms of the ITS region from (A) *C. bertholletiae* NCK234 and (B) *R. microsporus* NCK408, sequenced from ITS-D1/D2 amplicons generated using the ITS1/NL4 primer pair.

(A)

(B)

**Fig. S2**. Multiple sequence alignment of the ITS region of *Cunninghamella bertholletiae* isolates from this study (Cub-a-1-1 and Cub-b-1-1) and reference sequences retrieved from the NCBI Nucleotide database, showing length variation in six homopolymeric tracts, highlighted in yellow.
